# Supplementary material for: Associations between Frailty and Ambient Temperature in Winter: Findings from a Population-Based Study
Source: Int J Environ Res Public Health. 2022 Dec 28;20(1):513. doi: 10.3390/ijerph20010513 (PMC9819953; doi:10.3390/ijerph20010513)
Supplement: Supplementary file 1 [file ijerph-20-00513-s001.zip › ijerph-2012605-supplementary.pdf]

Supplementary Material

Table S1. List of items included in the Frailty Index

No.	Items
1	IADLs: Unable to visit neighbors by oneself
2	IADLs: Unable to shop by oneself if necessary
3	IADLs: Unable to cook meals by oneself if necessary
4	IADLs: Unable to wash clothing by oneself
5	IADLs: Unable to walk continuously for 1 kilometer
6	IADLs: Unable to lift a weight of 5 kg (such as a heavy bag of groceries)
7	IADLs: Unable to continuously crouch and stand up three times
8	IADLs: Unable to use public transportation
9	Functional limitations: Unable to put hand behind neck
10	Functional limitations: Unable to put hand behind lower back
11	Functional limitations: Unable to raise arm upright
12	Functional limitations: Unable to stand up from sitting in a chair
13	Functional limitations: Unable to pick up a book from the floor
14	ADLs: Needs assistance bathing
15	ADLs: Needs assistance dressing
16	ADLs: Needs assistance toileting
17	ADLs: Needs assistance in indoor transferring
18	ADLs: Needs assistance eating
19	ADLs: Incontinence
20	Cognitively impaired (based on the Mini Mental State Examination)
21	Poor self-rated health
22	Health worsened in the past year
23	Poor interviewer-rated health
24	Hearing loss
25	Vision loss
26	Abnormal heart rhythm
27	Symptom of psychological distress (based on loneliness, usefulness, and fearfulness)
28	Number of serious illnesses in the past 2 years
29	Suffering from hypertension
30	Suffering from diabetes
31	Suffering from tuberculosis
32	Suffering from heart disease
33	Suffering from stroke/cerebrovascular disease
34	Suffering from bronchitis, emphysema, asthma, or pneumonia
35	Suffering from cancer
36	Suffering from arthritis
37	Suffering from bedsores
38	Suffering from gastric or duodenal ulcers
39	Suffering from Parkinson's disease

Abbreviations: IADLs instrumental activities of daily living; ADLs activities of daily living.
Note: Item No. 28 was assigned a value of 2.

Table S2. Average temperature in January, FI, and age for the participants with or without subsequent surveys in the first survey

Variables	All participants	Participants with subsequent surveys	Participants without subsequent surveys		
			All	Death	Lost
	(N=21,602)	(N=9421)	(N=12,181)	(N=7883)	(N=4298)
FI, median (IQR)	0.17 (0.22)	0.12 (0.14)	0.24 (0.25)	0.27 (0.24)	0.17 (0.23)
Age (years), mean ± SD	87.71 ± 11.59	82.81 ± 11.32	91.51 ± 10.30	93.76 ± 8.61	87.38 ± 11.77

Abbreviations: N number; SD standard deviation; IQR interquartile range.

Table S3. Logistic regression analysis for average temperature in January and frailty in the first survey

Variables	Age adjusted OR (95%CI)		Fully adjusted OR (95%CI)	
	Pre-frail	Frail	Pre-frail	Frail
Quartiles of AT in January				
Q4	Reference	Reference	Reference	Reference
Q3	1.05 (0.95, 1.15)	1.26 (1.14, 1.40)***	0.99 (0.89, 1.09)	1.05 (0.93, 1.19)
Q2	1.26 (1.13, 1.39)***	1.89 (1.69, 2.10)***	1.22 (1.08, 1.37)**	1.51 (1.31, 1.74)***
Q1	1.25 (1.13, 1.39)***	2.23 (1.99, 2.50)***	1.32 (1.12, 1.55)***	1.81 (1.49, 2.20)***
1-unit decrease of AT in January	1.01 (1.00, 1.01)***	1.04 (1.03, 1.04)***	1.01 (1.00, 1.01)*	1.03 (1.02, 1.04)***

Abbreviations: AT in January, Average temperature in January; Q1 1st quartile; Q2 2nd quartile; Q3 3rd quartile; Q4 4th quartile; 95%CI 95% confidence interval; OR odds ratio.
Note: In the fully adjusted model, ORs were adjusted for age, sex, ethnicity, residence, current marital status, education, occupation, family income last year, smoking at the present, drinking at the present, exercising at the present, social and leisure activity index, yearly rainfall and geographic regions. Note that temperature change was not included in the fully adjusted model due to the high correlation with average temperature in January.
$^{***}p < 0.001$, $^{**}p < 0.01$, $^{*}p < 0.05$

Table S4. GEE analysis for the associations between average temperature in January and frailty

Variables	Age adjusted OR (95%CI)		Fully adjusted OR (95%CI)	
	Pre-frail	Frail	Pre-frail	Frail
Quartiles of AT in January				
Q4	Reference	Reference	Reference	Reference
Q3	1.23 (1.12, 1.35)***	1.28 (1.14, 1.44)***	1.17 (1.06, 1.29)**	1.14 (1.00, 1.30)
Q2	1.28 (1.16, 1.41)***	1.71 (1.51, 1.92)***	1.22 (1.09, 1.37)***	1.25 (1.08, 1.46)**
Q1	1.35 (1.22, 1.49)***	2.23 (1.97, 2.53)***	1.35 (1.15, 1.58)***	1.64 (1.33, 2.01)***

1-unit decrease of AT in January	1.01 (1.00, 1.01)***	1.03 (1.03, 1.04)***	1.01 (1.00, 1.02)*	1.02 (1.01, 1.03)***

Abbreviations: AT in January, Average temperature in January; Q1 1st quartile; Q2 2nd quartile; Q3 3rd quartile; Q4 4th quartile; 95%CI 95% confidence interval; OR odds ratio.

Note: In the fully adjusted model, ORs were adjusted for age, sex, ethnicity, residence, current marital status, education, occupation, family income last year, smoking at the present, drinking at the present, exercising at the present, social and leisure activity index, yearly rainfall and geographic regions. Note that temperature change was not included in the fully adjusted model due to the high correlation with average temperature in January.

$^{***}p<0.001$, $^{**}p<0.01$, $^{*}p<0.05$

Table S5. GLMM analysis for the associations between average temperature in January and frailty with original data

Abbreviations: AT in January, Average temperature in January; Q1 1st quartile; Q2 2nd quartile; Q3 3rd

Variables	Age adjusted OR (95%CI)		Fully adjusted OR (95%CI)	
	Pre-frail	Frail	Pre-frail	Frail
Quartiles of AT in January				
Q4	Reference	Reference	Reference	Reference
Q3	1.18 (1.13, 1.24)***	1.31 (1.22, 1.40)***	1.18 (1.12, 1.24)**	1.31 (1.21, 1.41)***
Q2	1.37 (1.30, 1.44)***	1.95 (1.82, 2.09)***	1.37 (1.29, 1.46)***	1.73 (1.58, 1.90)***
Q1	1.33 (1.26, 1.40)***	2.35 (2.19, 2.51)***	1.39 (1.28, 1.52)***	1.99 (1.76, 2.24)***
1-unit decrease of AT in January	1.01 (1.00, 1.01)***	1.04 (1.04, 1.05)***	1.01 (1.00, 1.02)*	1.03 (1.02, 1.04)***

quartile; Q4 4th quartile; 95%CI 95% confidence interval; OR odds ratio.

Note: In the fully adjusted model, ORs were adjusted for age, sex, ethnicity, residence, current marital status, education, occupation, family income last year, smoking at the present, drinking at the present, exercising at the present, social and leisure activity index, yearly rainfall and geographic regions. Note that temperature change was not included in the fully adjusted model due to the high correlation with average temperature in January.

$^{***}p<0.001$, $^{**}p<0.01$, $^{*}p<0.05$
